# COVID-19 on patients with immune-mediated rheumatic disease: a comparative study of disease activity, fatigue, and psychological distress over six months

**DOI:** 10.1186/s42358-025-00434-x

**Published:** 2025-01-22

**Authors:** Claudia Marques, Marcelo M. Pinheiro, Jennifer Lopes, Sandra Lúcia Euzébio Ribeiro, Mary Vânia Marinho de Castro, Lilian David de Azevedo Valadares, Aline Ranzolin, Nicole Pamplona Bueno de Andrade, Rafaela Cavalheiro do Espírito Santo, Nafice Costa Araújo, Cintya Martins Vieira, Valéria Valim, Flavia Patricia Sena Teixeira Santos, Laurindo Ferreira da Rocha, Adriana Maria Kakehasi, Ana Paula Monteiro Gomides Reis, Edgard Torres dos Reis-Neto, Gecilmara Salviato Pileggi, Gilda Aparecida Ferreira, Licia Maria Henrique da Mota, Odirlei Monticielo, Ricardo Machado Xavier

**Affiliations:** 1 Department of Rheumatology, Hospital das Clínicas da Universidade Federal de Pernambuco/Ebserh, Recife, Pernambuco Brazil; 2 Department of Rheumatology, Escola Paulista de Medicina, Universidade Federal de São Paulo (EPM/UNIFESP), São Paulo, Brazil; 3 Postgraduate Program of Translacional Health, Universidade Federal de Pernambuco, Recife, Brazil; 4 Department of Rheumatology, Faculdade de Medicina da Universidade Federal do Amazonas, Manaus, Amazonas Brazil; 5 Department of Internal Medicine, Hospital Getúlio Vargas, Recife, Pernambuco Brazil; 6 Department of Rheumatology, Hospital de Clínicas de Porto Alegre/Universidade Federal do Rio Grande do Sul, Porto Alegre, Rio Grande do Sul Brazil; 7 Department of Rheumatology, Hospital do Servidor Público Estadual de São Paulo - Instituto de Assistência Médica ao Servidor Público Estadual, São Paulo, Brazil; 8 Department of Rheumatology, Hospital Universitário da Universidade Federal de Juiz de Fora, Juiz de Fora, Minas Gerais Brazil; 9 Department of Rheumatology, Universidade Federal de Sergipe, Aracaju, Brazil; 10 Department of Rheumatology, Hospital das Clínicas - Universidade Federal de Minas Gerais, Belo Horizonte, Minas Gerais Brazil; 11 Department of Rheumatology, Instituto de Medicina Integral Professor Fernando Figueira, Recife, Pernambuco Brazil; 12 Department of Rheumatology, Centro Universitário de Brasília, Brasília, Brazil; 13 Department of Rheumatology, Universidade de Brasília, Brasília, Brazil

## Abstract

**Objectives:**

To compare the impact of COVID-19 on the clinical status and psychological distress of patients with immune-mediated rheumatic disease (IMRD) caused by SARS-CoV-2 infection with that of noninfected IMRD controls during a 6-month follow-up period.

**Methods:**

The ReumaCoV Brazil is a longitudinal study designed to follow IMRD patients for 6 months after COVID-19 (patients) compared with IMRD patients without COVID-19 (controls). Clinical data, disease activity measurements and current treatments regarding IMRD and COVID-19 outcomes were evaluated in all patients. Disease activity was assessed through validated tools at inclusion and at 3 and 6 months post-COVID-19. Fatigue, using FACIT-F (Functional Assessment of Chronic Illness Therapy) and psychological distress, using DASS 21 (Depression, Anxiety and Stress Scale − 21 Items), used to evaluated psychological distress, were evaluated at 6 months after COVID-19 in both groups. The significance level was set as *p* < 0.05, with a 95% confidence interval.

**Results:**

A total of 601 patients were evaluated—321 patients (IMRD COVID-19 + patients) and 280 controls (IMRD COVID-19- patients)—who were predominantly female with similar median ages. Disease activity assessment over a 6-month follow-up showed no significant difference between cases and controls. Although the mean activity scores did not differ significantly, some patients reported worsened disease activity post-COVID-19, particularly in rheumatoid arthritis (RA) (32.2%) and systemic lupus erythematosus (SLE) patients (23.3%). Post-COVID-19 worsening in RA patients correlated with medical global assessment (MGA) and CDAI scores, with a moderate to large effect size. Diabetes mellitus showed a positive association (OR = 7.15), while TNF inhibitors had a protective effect (OR = 0.51). Fatigue, depression, anxiety, and stress were significantly greater in patients than in controls. Worse disease activity post-COVID-19 correlated with worse FACIT-F and DASS-21 scores in RA patients. No significant associations were found between COVID-19 outcomes and post-COVID-19 disease activity, FACIT-F or DASS-21.

**Conclusions:**

Post-COVID-19 IMRD patients exhibited significant fatigue, depression, anxiety, and stress, which can be mistaken for disease activity, despite having similar disease activity scores. The variability in reports on IMRD flares and the potential triggering of SARS-CoV-2 for autoimmune manifestations underscore the need for detailed clinical assessment and a comprehensive approach to managing them.

## Introduction

The COVID-19 pandemic caused by the SARS-CoV-2 virus represents one of the greatest public health challenges worldwide. The infection has raised concerns about how the disease behaves in patients with immune-mediated rheumatic disease (IMRD), particularly in terms of flares after infection and immunosuppressive treatment as potential risk factors for severe COVID-19. Since 2020, much information has been published about this association and its implications, but few studies have evaluated rheumatic disease flares prospectively post-COVID-19 [1], and the existing data are still controversial.

Some individuals with immune-mediated rheumatic disease (IMRD) may experience exacerbation following SARS-CoV-2 infection. However, uncertainties persist regarding whether these exacerbations are directly linked to disease activity or represent manifestations associated with COVID-19 itself. The phenomenon commonly referred to as “long COVID” has been associated with a diverse range of clinical manifestations, encompassing joint pain, fatigue, and psychological symptoms such as depression and anxiety, which can persist for several months postinfection [2]. In patients with IMRD, the presence of these symptoms may be connected to disease flares, introducing ambiguity in determining the appropriate course of treatment. Additionally, some authors have highlighted psychosomatic aspects or a fibromyalgia-like presentation, particularly as the prevalence increased following the initial wave of the pandemic. This increase appears to be more strongly correlated with heightened fears, uncertainties, and lockdown strategies, as biological abnormalities such as impaired immune response or viral persistence have not yet been conclusively identified [2].

This study aimed to assess the disease activity status of patients with immune-mediated rheumatic disease (IMRD) who had experienced COVID-19, comparing them to those who did not contract the virus, over a 6-month follow-up period. Furthermore, the objective of this study was to delineate the frequence of fatigue, depressive symptoms, anxiety, and stress, and to investigate the potential association of these symptoms with IMRD flares.

## Materials and methods

### Study design – ReumaCoV Brazil cohort

This paper presents the protocol for the ReumaCoV-Brasil Registry protocol as described elsewhere [3]. Briefly, a prospective observational cohort study was carried out in 13 university centers distributed across all five Brazilian geographic regions, including patients with IMRD and COVID-19 and a comparison group (patients with only IMRD), with a follow-up time of 6 months. Patients were evaluated at three consecutive visits—visit 1 (inclusion), visit 2 (3 months) and visit 3 (6 months)^−from 2^ May to 31 December 2020.

Given the pandemic and compulsory social isolation, we are adding the possibility of the patient’s first interview being conducted via telephone contact. From the moment the patient contacted the attending physician reporting having presented symptoms suggestive of COVID-19, verbal consent was requested, the information was registered at REDCap, and the first interview was conducted by phone. Within two weeks after the first interview, the patient returned to the hospital for blood collection and a physical examination, and at this time, they signed the informed consent before these procedures were carried out. Disease activity status in the medical records of the most recent consultation, carried out at least in the last 6 months before inclusion, was considered pre-COVID status. The post-COVID status was defined as the status at which the patient presented at inclusion in the study.

### Disease activity evaluation

At all visits, data related to disease activity were collected using specific validated instruments, such as the Clinical Disease Activity Index (CDAI) [4] for RA patients, the Systemic Lupus Erythematosus Disease Activity Index 2000 (SLEDAI-2k) [5] for SLE patients, and the Bath Ankylosing Spondylitis Disease Activity Index (BASDAI) [6] for patients with SpA. In addition to the activity scores, medical global assessment (MGA) and patient global assessment (PGA) scores were collected regarding disease activity using a visual analog scale (VAS) from 0 to 10 points, with 10 being the best score, and the patient’s opinion regarding DRIM being the worst since the last visit, also using a VAS from 0 to 10.

### Disease flare definition

- Rheumatoid arthritis: worsening of symptoms compared to the pre-COVID-19 state plus an increase of 4.5 points in the CDAI score and/or need to change treatment [7].
- Systemic Lupus Erythematosus: worsening of symptoms compared to the pre-COVID-19 state plus an increase of at least 4 points in the SLEDAI and/or need to change treatment [8].
- Spondyloarthritis: worsening of symptoms compared to the pre-COVID-19 state plus an increase of at least 2 points in the BASDAI score and/or the need to change treatment [9].

### Psychological distress evaluation

At visit 3, two questionnaires were administered: one to assess fatigue, using the Functional Assessment of Chronic Illness Therapy – Fatigue Scale (FACIT-F) [10], and the other to assess mental symptoms, using the Depression, Anxiety and Stress Scale (DASS-21) [11].

The FACIT-F is a short, 13-item tool that measures an individual’s level of fatigue during their usual daily activities over the past week. Fatigue was measured on a four-point Likert scale (4 = not at all fatigued to 0 = very much fatigued). The score varies from 0 to 52, and the lower the result is, the greater the level of fatigue.

The DASS-21 is used as a quantitative measure of distress along the 3 axes of depression (D), anxiety (A) and stress (S) reactions and management. Each of the questions is rated from 0 to 3. Therefore, each of the axes presents partial scores of 0 to 18–24 depending on the number of questions assigned.

The study was registered at the Brazilian Registry of Clinical Trials RBR-33YTQC and approved by the Brazilian National Research Council (CONEP) under number 3.955.206. All participants were requested to provide explicit opt-in consent prior to participating in the survey.

### Statistical analysis

The Kolmogorov-Smirnoff test was performed to verify the normal distribution of continuous variables. As nonnormality was verified, the median was used as a measure of central tendency with the respective interquartile range (IQR).

To verify the associations among categorical or continuous variables, the chi-square or Fisher test and Pearson test were used, respectively. The Mann‒Whitney test was used to compare means and to estimate the effect size by calculating the r index and the R squared (R^2^). The R index was used to evaluate the correlation between two variables: small effect size: 0.10; medium effect size: 0.30; and large effect size: 0.50.

To determine the effect size (ES) for comparing means, Cohen’s d test was used, interpreted as follows: 0.2 to 0.3 for small effects (the difference between the groups is subtle); approximately 0.5 for medium effects (moderate difference between the groups); and 0.8 or more for large effects (substantial difference between the groups). To compare the variables across three visits, one-way ANOVA (95% CI) was used for repeated measures.

For this analysis, the SPSS statistical package, version 29.0.2.0, and GraphPad Prism software, version 10.1.1 (270), November 21, 2023, were used. The significance level was set at *p* < 0.05, with a 95% confidence interval.

## Results

A total of 601 patients were evaluated, including 321 patients (IMRD COVID+) and 280 controls (IMRD COVID-), with the majority being female with a similar median age. No differences in demographic data, including comorbidities, disease duration and IMRD, were observed between the two groups. A greater frequency of social isolation was observed in the control group (*p* = 0.001) and in the TNFi treatment group (*p* = 0.003). Table 1 summarizes the clinical and demographic data at baseline.

**Table 1:** Clinical and demographic data of the sample comparing IMRD patients with and without COVID-19 at baseline. The chi-square test was used unless otherwise noted

	Cases (IMRD COVID +) *n* = 321		Controls (IMRD COVID -) *N* = 280		*P* value (CI = 95%)
	*n*	%	*n*	%	
**Female**	275	85.7	237	84.6	0.724
**Age (median**,** IQR)**	46 (37–57)		49 (38–57)		0.136**
**Ethnicity**					
*White*	105	32.7	99	35.3	0.601
*Nonwhite*	216	67.3	181	64.7	
**Instruction**					
*Illiterate*	1	0.3	2	0.7	0.631
*< 8 years*	78	24.3	74	26.5	
*> 8 years*	242	75.4	204	72.8	
**Profession**					
*Public attendance*	41	12.8	33	11.7	**0.030**
*Health*	34	10.6	13	4.7	
*Security*	8	2.5	3	1.1	
*Education*	32	10.0	25	9.0	
*Housewife*	90	28.1	77	27.6	
*Others*	116	36.0	129	45.9	
**Active at work**	166	51.9	125	44.8	0.084
**Social isolation**	162	50.5	188	67.1	**0.001**
**Comorbidities**					
*Hypertension*	123	38.3	120	42.9	0.258
*Obesity*	48	15.0	34	12.1	0.317
*Diabetes*	31	9.7	22	7.9	0.438
*Lung disease*	20	6.2	16	5.7	0.790
*Kidney disease*	20	6.2	16	5.7	0.790
*Cardiopathy*	12	4.3	13	4.6	0.734
*No comorbidities*	107	33.3	99	35.4	0.602
**Smoking**	11	3.7	15	4.6	0.580
**Ethilism**	10	3.1	8	2.8	0.835
**Diagnosis**					
*SLE*	137	42.5	112	39.4	0.183
*RA*	93	28.9	105	37.0	
*Axial SpA*	23	7.1	30	10.6	
*Psoriatic arthritis*	21	6.5	12	36.4	
*Systemic sclerosis*	17	5.3	10	3.6	
*Sjögren disease*	12	3.7	5	1.8	
*Miopathy*	7	2.2	2	0.7	
Others	11	3.4	4	1.4	
**Disease duration**,** years** (median, IQR)	10 (5–15)		10 (6–18)		0.146**
**Medication IMRD**					
*Hydroxychloroquine*	139	43.3	113	40.4	0.465
*Oral corticosteroid*	137	42.7	109	38.9	0.351
*Methotrexate*	72	22.4	73	26.1	0.298
*TNFi*	61	19.0	82	29.3	**0.003**
*Azathioprine*	48	15.0	30	10.7	0.123
*Leflunomide*	36	11.2	23	8.2	0.217
*Mycophenolate mofetil*	28	8.7	24	8.6	0.948
*Tocilizumab*	14	4.4	23	8.2	0.050
*Rituximab*	5	1.6	6	2.1	0.762*
*IL-17i*	8	2.5	2	0.7	0.115*
*JAKi*	8	2.5	4	1.4	0.396*
*Abatacept*	4	1.2	2	0.7	0.690*
*Belimumab*	3	0.9	3	1.1	0.591*
*Cyclophosphamide (pulse therapy)*	3	0.9	3	1.1	1.000*
*Sulfasalazine*	3	0.9	6	2.1	0.316*

The chi-square test was used unless otherwise noted. *Fisher’s exact test; **Mann‒Whitney test; CI 95%. SLE: systemic lupus erythematosus; RA: rheumatoid arthritis; Axial SpA: axial spondyloarthritis; Pso arthritis: psoriatic arthritis; TNFi: tumor necrosis factor inhibitor; IL-17i: interleukin 17 inhibitor; JAKi: Janus kinase inhibitor; CI = Confidence interval

Among the patients with COVID-19 (*n* = 321), the most frequent symptoms were headache (60.6%), fever (55.6%), dysgeusia (54.3%), asthenia (53.7%), anosmia (52.5%) and cough (49.1%). Dyspnea was reported in 35.1% of patients. The medications used to treat COVID-19 were analgesics (53.7%), azithromycin (40.7%), oral corticosteroids (20.2%) and hydroxychloroquine (10.2%), but 16.1% did not use any medication.

Regarding COVID-19 outcomes, 77 (23.9%) patients sought hospital care, and 29 (9.0%) were hospitalized. Three of these patients were admitted to the intensive care unit and received mechanical ventilation. None of the patients died.

### Disease activity assessment

**Fig. 1:**
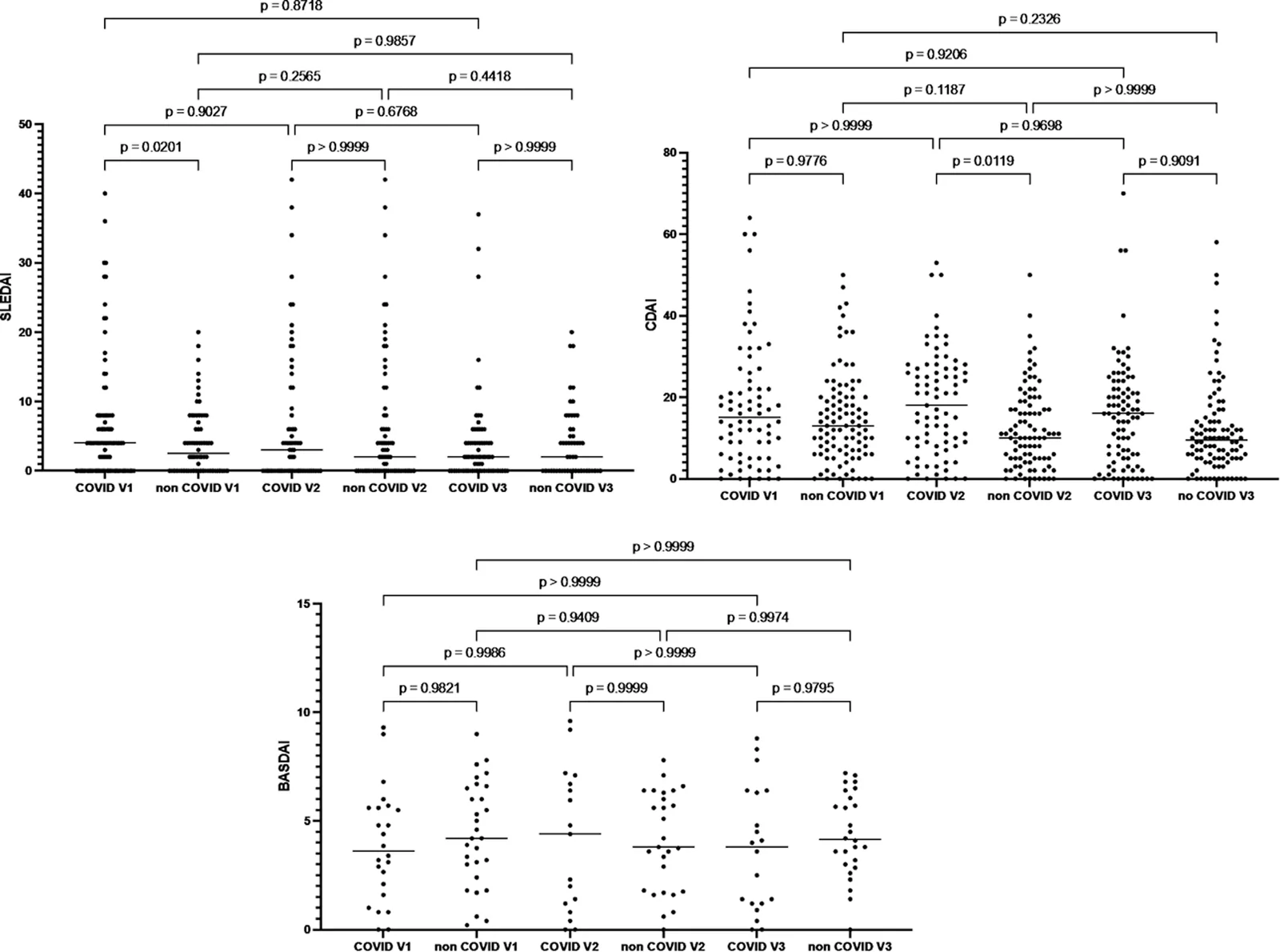
Comparison of disease activity scores across the three study visits (v1, V2 and V3) in patients with systemic lupus erythematosus, measured by the SLEDAI (**A**); rheumatoid arthritis, measured by the CDAI (**B**); and axial spondylarthritis patients, measured by the BASDAI, comparing cases and controls matched for sex, age and epidemiological exposure. V1: visit 1 (inclusion); V2: visit 2; V3: visit 3. CDAI: Clinical Disease Activity Score; SLEDAI: Systemic Lupus Erythematosus Disease Activity Index; BASDAI: Bath Ankylosing Spondylitis Disease Activity Index. One-way ANOVA (95% CI), repeated measures, multiple comparison of the mean of each column with the mean of every other column

There was no significant difference between the cases and controls at the 6-month follow-up (V1, V2, and V3), regardless of the IMRD (Fig. 1).

**Fig. 2:**
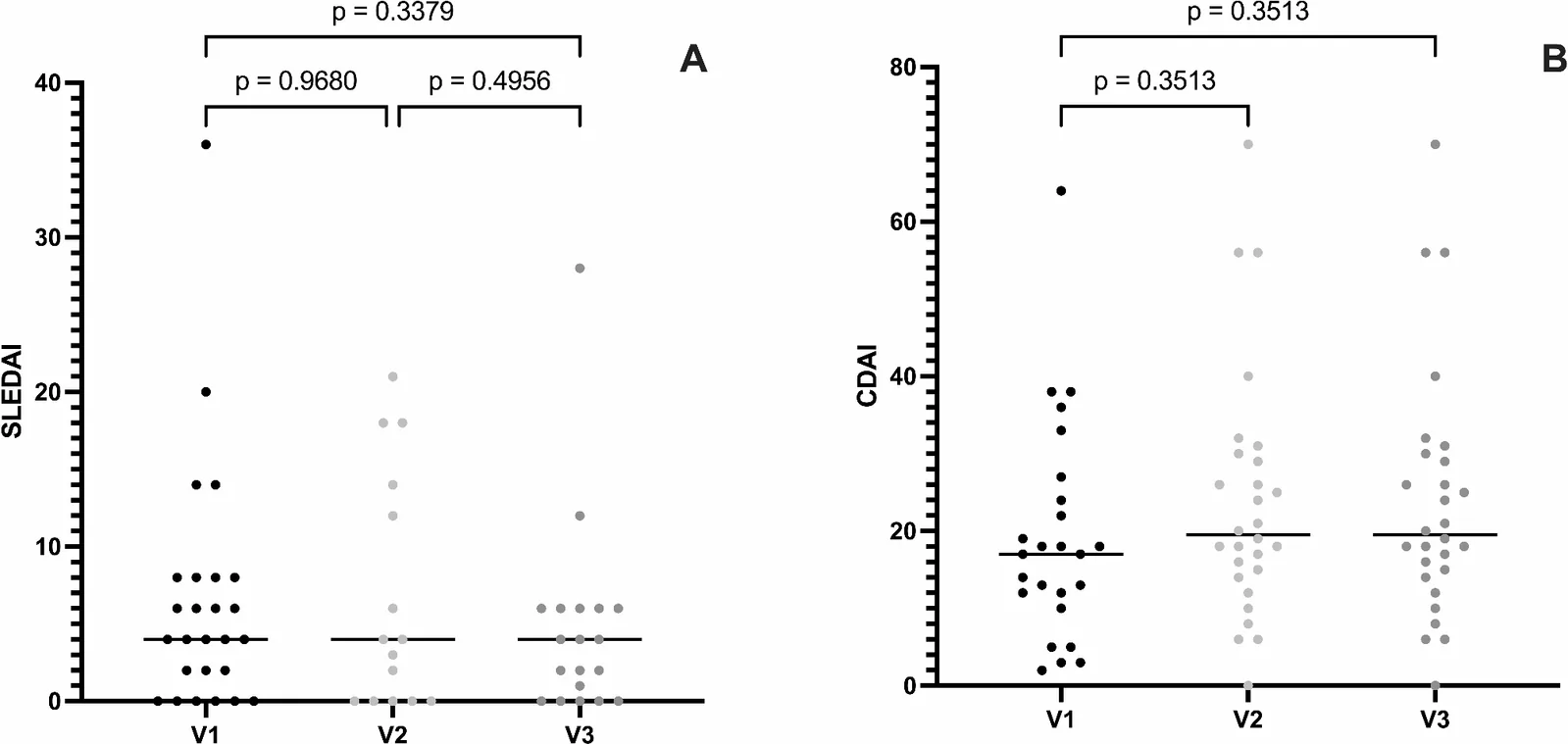
Comparison of disease activity scores across the three study visits in patients with systemic lupus erythematosus (SLEDAI) (**A**) and rheumatoid arthritis (CDAI) (**B**) who self-reported clinical worsening after COVID-19. Visit 1 (inclusion); V2: visit 2; V3: visit 3. CDAI: Clinical Disease Activity Score; SLEDAI: Systemic Lupus Erythematosus Disease Activity Index. One-way ANOVA (95% CI), repeated measures, multiple comparison of the mean of each column with the mean of every other column

Although no difference was demonstrated in the mean activity scores, some patients self-reported worsening of disease activity after COVID-19 [30 with RA (32.2%), 32 with SLE (23.3%) and 2 with SpA (8.6%)]. Furthermore, we did not observe any significant difference in the mean activity score between the RA and SLE patients (Fig. 2).

In RA patients who self-reported worsening of disease activity after COVID-19, we applied the flare definition, comparing the pre-COVID-19 CDAI score with the post-COVID-19 CDAI score (inclusion), visit 2 and visit 3, in a paired way. At inclusion, 12 patients (40.0%) had an increased CDAI ≥ 4.5 points compared to their pre-COVID status, but only 1 patient required a change in treatment. At visit 2, compared with visit 1, 5 new patients presented an increased CDAI ≥ 4.5 points, and 7 patients improved compared to the previous visit. At visit 3, 12 patients presented an increased CDAI, and of these, 8 new patients did not worsen at visit 2, and only one needed a change in treatment.

In addition, we compared the tender joint count (TJC), swollen joint count (SJC), patient global assessment (PGA) and medical global assessment (MGA) across the three visits, and no significant differences were observed. Eight patients in this group (26.6%) self-reported worsening of joint manifestations.

Comparing the disease activity in the group of patients with RA who self-reported worsening with that in the group of patients who did not, there was an association with post-COVID-19 MGA, assessed at baseline (ES = − 0.56; CI95% -1.01 to -0.11, *p* = 0.007), and with the CDAI and all its components at visit 3 (Table 2).

**Table 2:** Comparison between pre- and post-COVID-19 mean scores in patients with RA who reported worsening and those who did not at visit 3

	Worse in visit 3				*p* value (CI = 95%)	Independent sample effect size (ES)*
	No		Yes			
	Mean	SD	Mean	SD		
CDAI pre-COVID^#^	17.5	16.2	19.2	14.1	0.330	-0.110 (-0.59 to 0.37)
TJC pre-COVID	5.9	6.8	6.4	5.5	0.369	-0.079 (-0.53 to 0.38)
SJC pre-COVID	3.6	6.0	3.3	5.6	0.404	0.057 (-0.40 to 0.51)
PGA pre-COVID	4.8	3.0	8.6	16.4	0.055	-0.385 (-0.85 to 0.087)
MGA pre-COVID	3.2	2.7	3.8	2.6	0.210	-0.196 (-0.66 to 0.27)
CDAI post-COVID^&^	19.0	18.4	26.5	19.3	0.040	-0.397 (-0.83 to 0.04)
TJC post-COVID	6.5	8.4	9.7	8.6	0.044	-0.384 (-0.82 to 0.05)
SJC post-COVID	3.1	4.8	4.8	7.0	0.113	-0.271 (-0.70 to 0.16)
PGA post-COVID	5.5	3.1	6.4	2.9	0.090	-0.304 (-0.74 to 0.140)
MGA post-COVID	3.4	2.9	5.0	2.7	**0.007**	**-0.563 (-1.01 to -0.11)**
CDAI V3	11.7	9.6	23.6	15.9	**0.001**	**-0.996 (-1.45 to -0.51)**
TJC V3	3.5	3.6	7.5	6.9	**< 0.001**	**-0.801 (-1.25 to -0.34)**
SJC V3	1.3	2.2	4.7	6.3	**0.001**	**-0.818 (-1.27 to -0.36)**
MGA V3	2.7	2.3	5.0	2.2	**0.001**	**-1.003 (-1.46 to -0.53)**
PGA V3	4.2	2.7	6.8	2.3	**0.001**	**-1.005 (-1.45 to -0.53)**

CDAI: clinical disease activity score; TJC: tender joint count; SJC: swollen joint count; PGA: patient global evaluation; MGA: medical global evaluation. *Cohen’s d test (95% CI): 0.2 to 0.3: Small effect - the difference between the groups is subtle. Approximately 0.5: Medium effect - moderate difference between the groups. 0.8 or more: Large effect - substantial difference between the groups; CI = Confidence interval

The size effect for MGA post-COVID-19 (V1) mean difference means was moderate (-0.56; CI95% -1.01 to -0.11), and for CDAI (-0.996; CI95% -1.45 to -0.51), TJC (-0.801; CI 95% -1.25 to -0.34), SJC (-0.818; CI-1.27 to -0.36), MGA (-1.003; CI -1.46 to -0.53) and PGA at V3 (-1.005; CI -1.45 to -0.53) was a large effect, demonstrating substantial differences between the groups.

Post-COVID-19 worsening in patients with RA was associated with diabetes mellitus (OR = 7.15; 95% CI 1.7–29.4, *p* = 0.005) and protection from the current use of TNF inhibitors before SARS-CoV-2 infection (OR = 0.51; 95% CI 0.2–0.9, *p* = 0.026).

Among the patients with SLE who self-reported worsening of disease after COVID-19, 10 reported the appearance of new clinical manifestations. However, no significant differences were observed regarding sex, age, comorbidities, or concomitant medications. Worsening was only associated with anosmia, a symptom of COVID-19 (OR = 2.43; 95% CI = 1.07–5.52, *p* = 0.03). The self-reported clinical manifestations of the SLE patients who were worse after COVID-19 (*n* = 32) at visit 3 were skin rash (5 patients), arthritis (3 patients), proteinuria (2 patients) and interstitial lung disease (1 patient).

By comparing the pre- and post-COVID-19 SLEDAI scores according to the flare definition, we observed that 6 patients (18.0%) had increased SLEDAI scores ≥ 4.0 points, but only 2 patients required a change in treatment. At visit 2, compared with visit 1, most patients showed improvement in the SLEDAI score, and only 3 patients experienced improvement in the SLEDAI score at visit 3. However, 3 new patients had increased SLEDAI scores compared with those at visit 2.

Among SpA patients, only 2 patients self-reported worsening after COVID-19, and one of them experienced progressive worsening at all three visits, based on the BASDAI.

There was no statistically significant association between COVID-19 outcomes (hospital care, hospitalization, and ICU admission) and post-COVID-19 disease activity at visit 3.

### Fatigue, depression, anxiety, and stress assessment

**Fig. 3:**
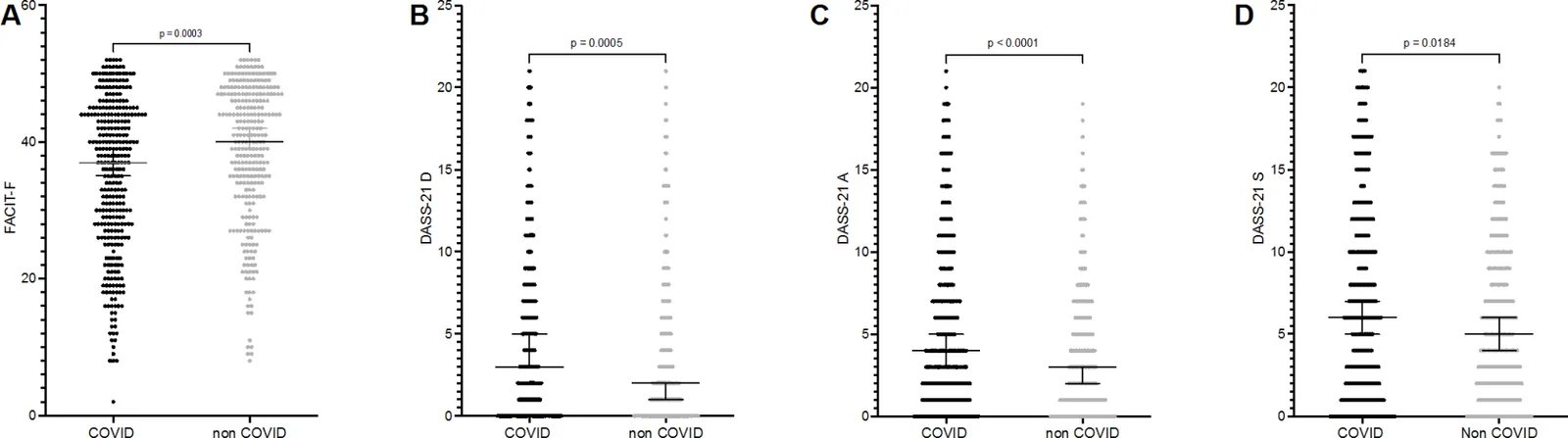
Comparisons among the FACIT (**A**), DASS-21D (**B**), DASS-21 A (**C**) and DASS-21 S (**D**) median (IQR) scores in patients and controls at visit 3. IQR: Interquartile range. FACIT-F: Functional Assessment of Chronic Illness Therapy–Fatigue; DASS-21D: Depression Anxiety and Stress Scale–Dominium Depression; DASS-21 A: Depression Anxiety and Stress Scale–Dominium Anxiety; DASS-21 S: Depression Anxiety and Stress Scale–Dominium Stress. Mann‒Whitney test; CI = 95%

After a 6-month follow-up, a statistically significant difference was observed in the FACIT and DASS-21 scores in the three domains between the cases and controls (Fig. 3).

**Fig. 4:**
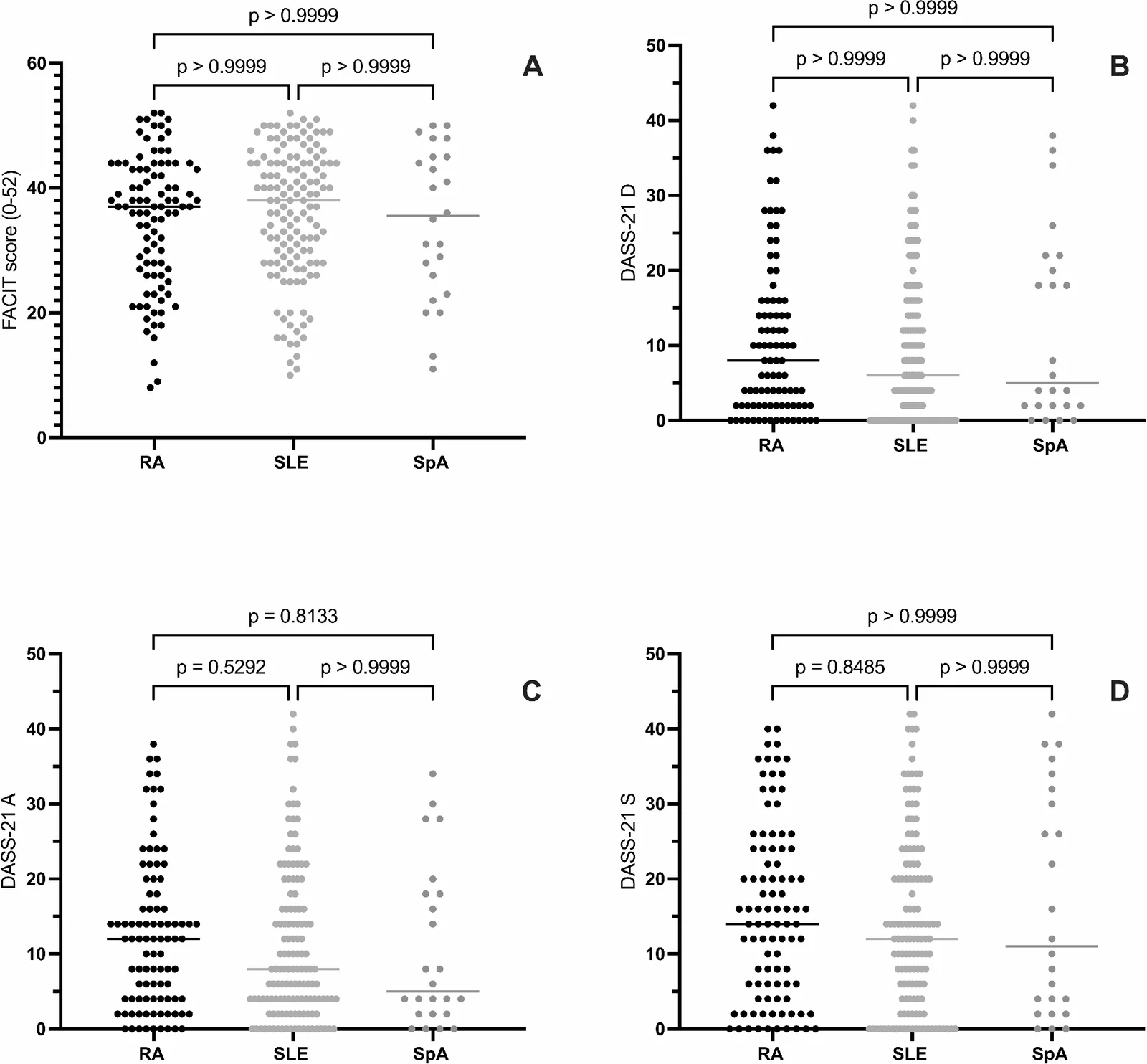
Distribution and comparison of FACIT (**A**), DASS21D (**B**), DASS21A (**C**) and DASS21S (**D**) median scores after a 6-month follow-up in patients with rheumatoid arthritis (RA), systemic lupus erythematosus (SLE) and axial spondyloarthritis (axSpA) only in the patient group (IMRD COVID-19 +). FACIT-F: Functional Assessment of Chronic Illness Therapy – Fatigue; DASS-21D: Depression Anxiety and Stress Scale Domain Depression Scale; DASS-21 A: Depression Anxiety and Stress Scale Domain Anxiety Scale; DASS-21 S: Depression Anxiety and Stress Scale Domain Stress Scale; PsoA: psoriatic arthritis; RA: rheumatoid arthritis; axSpA: axial spondyloarthritis; SLE: systemic lupus erythematosus. Independent-samples Kruskal‒Wallis test, 95% CI

The median FACIT score was 37 (27–44) in cases and 40 (32–47) in controls (*p* = 0.0003); the median DASS-21 D in cases was 3 (0–8) and 2 (0–5) in controls (*p* = 0.0005); the median DASS-21 A in cases was 4 (1–9) and 3 (1–6) in controls (*p* < 0.001); and the median DASS-21 S in cases was 6 (2–12) and 5 (2–9) in controls (*p* < 0.018). No difference was observed regarding the FACIT-F and DASS-21 scores when comparing patients with RA, SLE and SpA in each case group (Fig. 4).

**Fig. 5:**
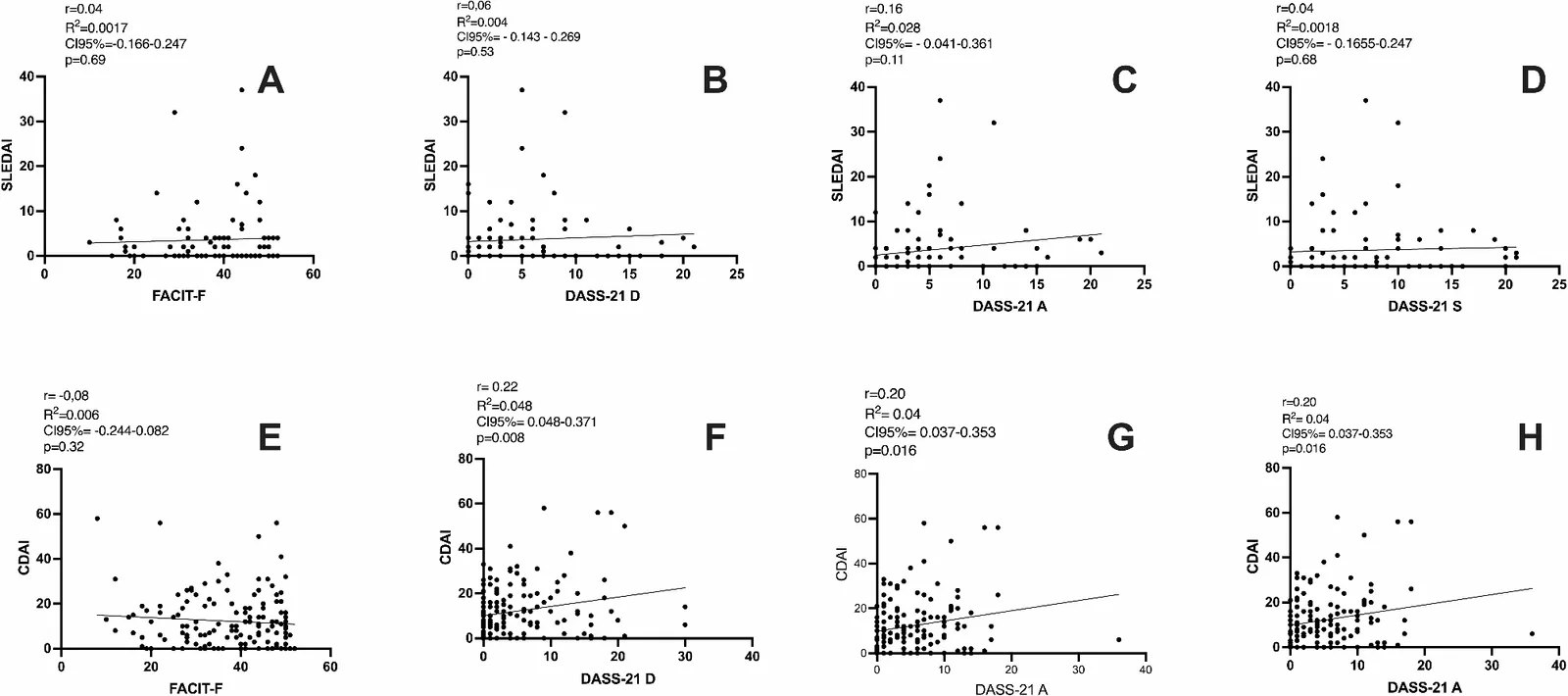
Correlation between disease activity in patients with systemic lupus erythematosus, as measured by the SLEDAI, and rheumatoid arthritis, as measured by the CDAI, only in the case group (IMRD COVID-19 +), with FACIT-F (**A** and **E**) and DASS 21-D (**B** and **F**), DASS 21-A (**C** and **G**) and DASS 21-S (**D** and **H**) scores

Univariate linear models revealed no associations between FACIT or DASS-21 and comorbidities, IMRD endpoints (type and specific therapy), or outcomes related to COVID-19, such as symptoms and treatment. Considering the FACIT-F and DASS-21 scores, there was no significant correlation between disease activity in RA patients and that in SLE patients in the case group (Fig. 5).

Specifically, when we analyzed the group of patients who experienced worsening of disease activity after COVID-19, we observed that RA patients also experienced a decrease in FACIT-F (31.5 vs. 36.4; *p* = 0.047) and DASS-21-S (19.0 vs. 13.0; *p* = 0.031) scores compared to those with no worsening activity. Additionally, the means were considered moderate for FACIT-F (0.47; from 0.03 to 0.91) and DASS 21-S (-0.44; from − 0.88 to -0.007) (Table 3). In SLE patients, no significant difference was observed.

**Table 3:** Comparison of the mean FACIT, DASS 21-D, DASS 21-A and DASS-21 S scores between patients with RA who reported worsening and those who did not

	Worse at visit 3 (RA patients)				*p* value (CI = 95%)	Independent sample effect size*
	No		Yes			
	Mean	SD	Mean	SD		
FACIT	36.4	9.7	31.5	11.3	**0.017**	**0.47 (0.03 to 0.91)**
DASS 21-D	9.5	10.5	12.6	10.9	0.094	-0.29 (-0.73 to 0.144)
DASS 21-A	11.3	10.0	14.4	9.9	0.090	-0.30 (-0.73 to 0.13)
DASS 21-S	13.7	12.4	19.0	9.7	**0.023**	**-0.44 (-0.88 to -0.007)**

*Cohen’s d (95% CI): 0.2 to 0.3: Small effect - the difference between the groups is subtle. Approximately 0.5: Medium effect - moderate difference between the groups. 0.8 or more: Large effect - substantial difference between the groups; CI = Confidence interval

There was no statistically significant association between COVID-19 outcomes (hospital care, hospitalization, or ICU admission) and FACIT-F or DASS-21 scores at visit 3.

## Discussion

Our results demonstrated that the disease activity scores did not differ significantly between IMRD patients during a 6-month follow-up after COVID-19 and those who did not contract the virus. However, clinical worsening was related to fatigue, depression, anxiety, and stress.

Currently, the data regarding the association between SARS-CoV-2 infection and the incidence of IMRD flare are controversial, especially because the published studies have heterogeneous samples and different designs. Consequently, the current uncertainty revolves around whether patient-reported outcomes (PROs) are related to the IMRD flare-up itself after COVID-19, whether it is associated with long COVID-19 or, alternatively, whether a reactive postinfectious immunological response is a potential trigger [1, 12–15].

In rheumatic diseases, a flare is defined as any exacerbation of disease activity that, if persistent, would typically necessitate the initiation or modification of therapy. It signifies a cluster of symptoms of sufficient duration and intensity to warrant the commencement, alteration, or escalation of therapeutic measures [16]. In our study, although there was no discernible difference in the mean activity scores among patients with rheumatoid arthritis (RA), systemic lupus erythematosus (SLE), or spondyloarthritis (SpA), a subgroup of patients reported a deterioration in immune-mediated rheumatic disease (IMRD). In some of these cases, activity scores increased, aligning with the flare definition employed in this investigation. Given the recognized potential for overestimation of IMRD activity in patients with conditions such as fibromyalgia [17] and other chronic painful disorders, particularly in RA and axial SpA, it is imperative to ascertain the presence of “true” disease activity. This determination holds substantial relevance in decision-making processes related to treatment adjustments. Nevertheless, we observed an association between patients’ self-reported worsening after 6 months and the Clinical Disease Activity Index (CDAI) score, along with all its individual components, including swollen joint count (SJC). This association suggests a plausible occurrence of flares in this specific subset of patients.

A cross-sectional study including 32 patients with RA and SpA demonstrated no relevant changes in disease activity after COVID-19 in those who interrupted their treatment [1]. According to data from the COVID-19 study, which evaluated 824 patients with IMRD who had at least one SARS-CoV-2 infection between March 2021 and June 18, 2022 [12], 36.9% of patients experienced at least one flare of the underlying IMRD following COVID-19 infection over a 127-day period (IQR = 62–308 days) from the date of infection to the date of the survey. Females and patients with comorbidities had greater odds of flaring of their disease. Patients who reported flares had worse physical health scores, pain VAS scores, fatigue VAS scores, and lower mental health scores than did those who did not report flares. However, as stated by the authors, “this was a self-reported disease flares without verification by a physician which could be impacted by patients’ perceptions of flares and their inability to distinguish from ongoing symptoms of long-COVID syndrome or secondary fibromyalgia”.

In a study of 92 children with IMRD who had COVID-19 [13], 10% experienced IMRD relapse after infection. Relapse was mild in four patients and moderate in five patients. One patient experienced severe relapse of ARD and required hospitalization, which was not associated with COVID-19 clinical presentation.

Similar to several other viral infections, SARS-CoV-2 infection couldpotentially trigger reactive and autoimmune diseases by inducing type II and type IV hypersensitivity reactions, leading to autoantibody production and autoimmune disease development as long-term complications [18], and in patients with pre-existing IMRD, it may be difficult to identify whether it is a flare of the disease or a postinfection manifestation. A recent study evaluated the effects of COVID-19 on the development and progression of RA using a collagen-induced arthritis (CIA) animal model. The incidence and severity of RA in CIA mice were slightly increased by the SARS-CoV-2 spike protein in vivo. In addition, the levels of autoantibodies and thrombotic factors, such as anti-CXC chemokine ligand 4 (CXCL4, also called PF4) antibodies and anti-phospholipid antibodies, were significantly increased by the SARS-CoV-2 spike protein. Furthermore, tissue destruction and inflammatory cytokine levels in joint tissue were markedly increased in CIA mice by the SARS-CoV-2 spike protein, suggesting that COVID-19 accelerates the development and progression of RA by increasing inflammation, autoantibody production, and thrombosis [19].

In an Italian study, 122 consecutive post-COVID-19 patients were evaluated, with the onset of rheumatic manifestations within 4 weeks of SARS-CoV-2 infection as an inclusion criterion. In this group, most patients had inflammatory joint disease (52.5%); 19.7% of patients were diagnosed with connective tissue diseases, and 6.6% of patients had vasculitis. Interestingly, in this same cohort, patients with inflammatory manifestations post-COVID-19 vaccination were evaluated, and this group had a greater percentage of patients classified as having polymyalgia rheumatica (PMR, 33.1% vs. 21.3%, *p* = 0.032) [14]. Another study evaluated the presence of arthritis associated with COVID-19 by ultrasound in 10 patients with (*n* = 4) and without previous rheumatic disease (*n* = 6). In the group without previous disease, 4 of the 6 patients presented with arthritis for 4 to 16 weeks after infection, comparable to reactive arthritis. One patient developed late-onset rheumatoid arthritis. In the group with previous disease, synovitis and tenosynovitis with positive Doppler power were observed, suggesting a possible flare-up of the disease after COVID-19 [20].

A major concern when interpreting musculoskeletal symptoms in these patients is the possibility of developing post-COVID syndrome, also known as long COVID-19. More than 20% of subjects surviving acute COVID-19 may suffer from persistent symptoms and develop new symptoms after one month, and approximately 5% of all infected individuals develop long-term complications after 6 months, possibly due to tissue damage, viral reservoirs, autoimmunity, and persistent inflammation [21, 22]. The clinical presentation in these patients, such as fatigue and joint pain, may mimic a disease flare, thereby complicating decision-making regarding treatment changes.

A recent systematic review revealed that the prevalence of arthralgia ranges from 2 to 65% within a time frame varying from 4 weeks to 12 months after COVID-19. Inflammatory arthritis has been reported to have various clinical phenotypes, including an RA-like pattern similar to that of other prototypical viral arthritis, as well as polymyalgia-like or acute monoarthritis and oligoarthritis of large joints resembling reactive arthritis or SpA [23].

An Iranian study evaluated the prevalence of musculoskeletal symptoms in 239 patients after the acute phase of COVID-19 and its associated factors using an online questionnaire. Almost all of them (98.74%) experienced at least one musculoskeletal symptom after recovering from COVID-19, and the most common symptom was fatigue (91.2%), followed by myalgia, headache, and low back pain. A high BMI, hospitalization, and ICU admission were associated with a greater risk of musculoskeletal symptoms [24].

In addition to musculoskeletal symptoms, patients could develop psychological distress after COVID-19, such as depression, anxiety, and stress, symptoms that could also be present in IMRD. The frequency of these symptoms in IMRD patients post-COVID-19 was significantly greater than that in patients who did not have the infection. Some authors have suggested that the term long COVID-19 is inappropriate and should be replaced by fibromyalgia-like post-COVID-19 syndrome [2].

We acknowledge the limitations of our study, such as the fact that we obtained FACIT and DASS-21 scores only at the last visit and that there was no possibility of comparison with the pre-COVID-19 status. Since the study began in the first months of the pandemic, we do not yet know that these outcomes were important. We decided to include it after the first studies demonstrating the association of these symptoms with SARS-CoV-2 infection were published. However, we know that fatigue, depression, anxiety, and stress are frequent symptoms in patients with IMRD, and we have a group that did not have COVID-19 as a comparator; in the group with COVID-19, these scores were significantly worse. In addition, the greater political and economic instability experienced in our country may have had a direct impact on psychological distress as a bias. On the other hand, we included a representative sample from various regions of Brazil, with a follow-up period of 6 months, compared with a group of patients with IMRD matched by sex and age for the same epidemiological period, which was one of the main strengths of our study.

## Conclusions

Our observational data highlight the complex interplay between COVID-19 and immune-mediated rheumatic disease (IMRD). Although disease activity scores may not show significant differences, individuals with post-COVID-19 IMRD may experience symptoms such as fatigue, depression, anxiety, and stress that can mimic disease activity and potentially lead to incorrect therapeutic decisions. The variations in reports regarding IMRD flares and the potential triggering role of SARS-CoV-2 in autoimmune manifestations further emphasize the necessity for thorough clinical assessment and a comprehensive approach to their management.

## Data Availability

The datasets during and/or analysed during the current study available from the corresponding author on reasonable request.
